# Survival of the glycosylated

**DOI:** 10.7554/eLife.65719

**Published:** 2021-01-22

**Authors:** Harry C Blair, Paul H Schlesinger

**Affiliations:** 1 Veterans Affairs Medical Center and the Department of Pathology, University of PittsburghPittsburghUnited States; 2 Department of Cell Biology and Physiology, Washington University School of MedicineSt LouisUnited States

**Keywords:** osteocalcin, glycosylation, bone hormones, osteoclasts, bone, plasmin, Human, Mouse

## Abstract

Osteocalcin is a bone matrix protein that acts like a hormone when it reaches the blood, and has different effects in mice and humans.

**Related research article** Al Rifai O, Julien C, Lacombe J, Faubert D, Lira-Navarrete E, Narimatsu Y, Clausen H, Ferron M. 2020. The half-life of the bone-derived hormone osteocalcin is regulated through *O*-glycosylation in mice, but not in humans. *eLife* **9**:e61174. doi: 10.7554/eLife.61174

Bone tissue is constantly being created and replaced in a process called remodeling. This involves cells called osteoblasts making bone and others cells called osteoclasts taking up the various minerals and proteins released from the degraded bone and discharging them into the blood (Figure 1). Osteocalcin is a protein found in bone tissue and is abundantly present in amounts equimolar to collagen – the structural protein of bone. Once absorbed by osteoclasts, osteocalcin displays variable carboxylation (the adding of a carboxyl acid group to the protein sequence) and is cleaved at various sites during its release.

**Figure 1.**
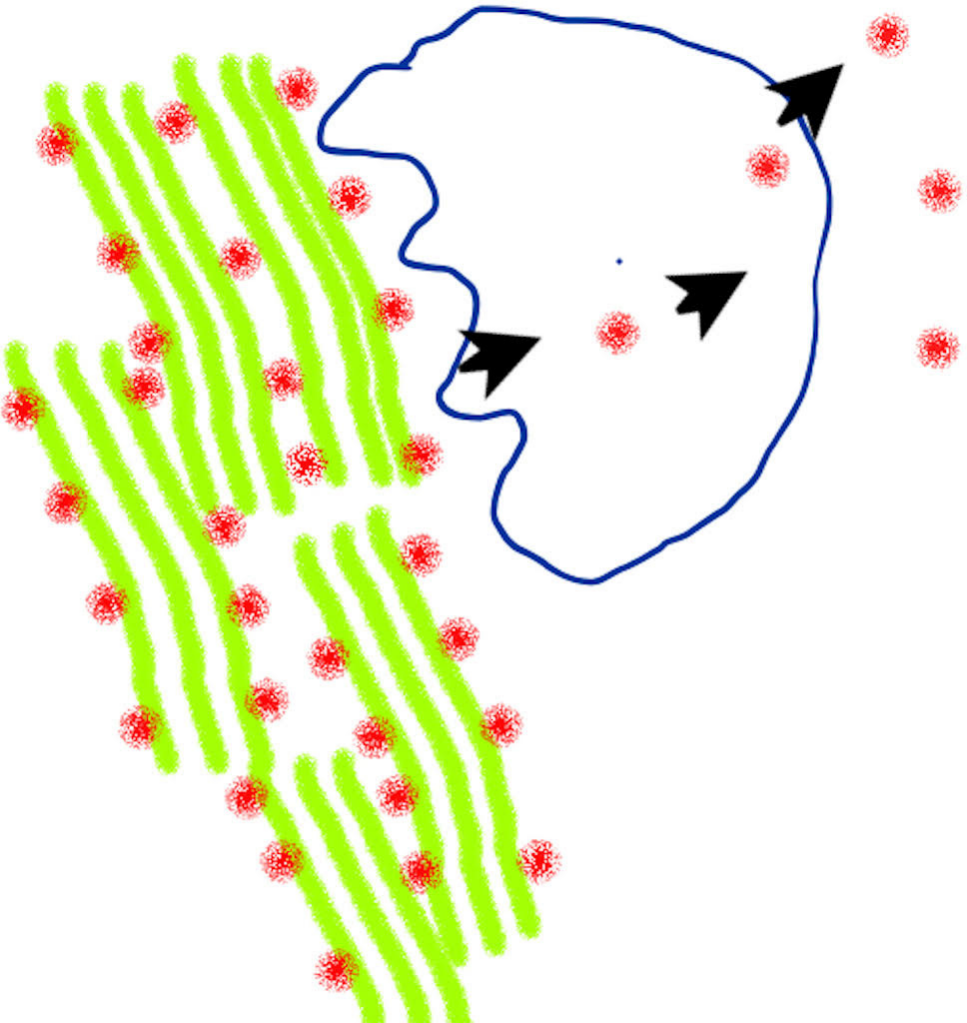
How osteocalcin is synthesized and released into the blood. The protein osteocalcin (red dots) resides in the bone matrix with collagen (green) and other matrix proteins integrated into mineralized bone. Bone tissue is broken down by cells called osteoclasts (shape outlined in blue), and left-over proteins – including osteocalcin – are partially degraded and discharged into the blood.

The osteocalcin molecules that reach the blood are a complex family of distinctive small peptides, most of which are less than 1000 kDa in weight (Blair et al., 1986). The family of discharged peptides include mixtures of intact or cleaved osteocalcin (Ivaska et al., 2004) and partially uncarboxylated protein (Poser and Price, 1979; Cairns and Price, 1994). These molecules behave like hormones, traveling to other organs in the body – such as the pancreas and liver – where they help to regulate metabolic processes (Tsuka et al., 2015).

It is widely posited that bone acts as endocrine organ that contributes to metabolic regulation (Dirckx et al., 2019). For example, knocking out the gene for osteocalcin in mice has been shown to impair glucose tolerance, to lead to increase bone formation, and to reduce testosterone production and muscle mass. However, these metabolic changes are variable and do not occur in all mouse models (Komori, 2020).

The G-protein receptor that osteocalcin binds to can also interact with numerous other ligands, such as calcium and steroids, which may be responsible for these differing observations (Pi et al., 2005). Furthermore, other hormones that bind to G-protein receptors, such as follicle stimulating hormone, have been found to activate receptors not on their target organ, and cause additional, unexpected effects (Sun et al., 2006). Together, this demonstrates the challenges associated with studying the function of osteocalcin, and why there is still a great deal to learn about the various roles of osteocalcin in mammals, including humans.

To coordinate metabolism in diverse tissues, the modified osteocalcin needs to be maintained at high levels in the blood and not be immediately removed by the kidney’s filtration system and discharged in the urine. Now, in eLife, Mathieu Ferron and co-workers from the Montreal Clinical Research Institute, the University of Montreal, the University of Copenhagen and McGill University – including Omar Al Rifai as first author – report how osteocalcin in mice is able to survive for longer in the blood than human osteocalcin (Al Rifai et al., 2020).

The team found that mouse osteocalcin undergoes an additional modification, which attaches a small sugar group called an O-glycan to a serine residue in the protein. Al Rifai et al. discovered that this sugar group prevented osteocalcin from being degraded by enzymes in the blood. Human osteocalcin was found to have a tyrosine residue at this site: however, when this was replaced with a serine residue, O-glycan groups were able to bind to the hormone and make it more stable, causing it to persist for longer in the blood. This difference in stability is likely what causes mice to have 5–10 times more osteocalcin in their blood than humans.

Much of what is known about the influence of osteocalcin on metabolism has come from experiments on mice. However, in addition to having different levels of osteocalcin, the molecular biology of this hormone also differs between humans and mice. In rodents, osteocalcin is coded by a cluster of three genes, two of which are expressed in osteoblasts (Moriishi et al., 2020), whereas human osteocalcin is coded by a single gene (Celeste et al., 1986).

Further work is needed to address other aspects of how osteocalcin is processed and regulated in humans. Previous studies suggest that levels of human osteocalcin are also regulated by the rate at which osteoclasts break down the bone’s tissue, and inhibiting this process has been found to reduce the amounts of osteocalcin in the blood by 30% (Zhou et al., 2020). However, the degradation of bone might not be the sole source of osteocalcin in humans (Figure 1), and some of our experiments suggest that when levels of osteocalcin are low, cells other than osteoblasts may also release moderate amounts of decarboxylated osteocalcin into the blood; but this remains to be established. This suggestion is supported by the fact that while decarboxylation in osteoclasts has not been fully studied, the process has been shown to be acid driven in vitro, and therefore unlikely to occur in osteoclasts (Poser and Price, 1979).

The findings of Al Rifai et al. demonstrate the challenges involved in extrapolating results about hormones and metabolism from mice to humans. Further work is still needed to fully understand the role of osteocalcin in humans, and to gain a clearer understanding of how levels of this hormone are regulated.
